# Usefulness of Positron Emission Tomography in the Examination of Hilar and Mediastinal Lymphadenopathies in Patients with Suspicion of Lung Cancer

**DOI:** 10.1155/2020/7909543

**Published:** 2020-06-05

**Authors:** Tara Pereiro-Brea, Antonio Golpe-Gómez, Antonio Miguel Golpe-Sánchez, Luís Valdés, Anxo Martínez de Alegría, José Martín Carreira-Villamor, Alberto Ruano-Raviña

**Affiliations:** ^1^Complejo Hospitalario Universitario A Coruña, Servicio de Neumología, A Coruña, Spain; ^2^Complejo Hospitalario Universitario Santiago de Compostela, Servicio de Neumología, Santiago de Compostela, Spain; ^3^Escuela de Medicina, Universidad de Santiago de Compostela, Área de Medicina Preventiva y Salud Pública, Santiago de Compostela, Spain; ^4^Grupo Interdisciplinario de Investigación en Neumología, Instituto de Investigaciones Sanitarias (IDIS), Santiago de Compostela, Spain; ^5^Complejo Hospitalario Universitario Santiago de Compostela, Servicio de Radiología, Santiago de Compostela, Spain; ^6^CIBER de Epidemiología y Salud Pública (CIBERESP), Madrid, Spain

## Abstract

**Introduction:**

Lung cancer is a major health problem. Mediastinal staging performed with the aid of imaging techniques is essential for appropriate disease treatment and prognosis. Accordingly, this study aimed to ascertain the usefulness of positron emission tomography (PET) in mediastinal staging, establish the best maximum standardized uptake value (SUV_max_) cutoff point, compare its usefulness to that of computed tomography (CT), and determine the influence of histological tumour subtype.

**Methods:**

We conducted a retrospective study across a period of 3 years on 128 patients with suspicion of lung cancer and analyzed their demographic and radiological characteristics using CT and PET to perform the mediastinal examination. Histology was regarded as the gold standard.

**Results:**

PET displayed a high sensitivity (95%) and negative predictive value (NPV) (92%), outperforming CT (89% and 85%, respectively). Percentage agreement with histology was also higher (0.207 and 0.241 for CT and PET, respectively; *p* < 0.001). Taking an SUV_max_ value of 0.5 as that which would ensure greatest diagnostic accuracy, S and NPV were 100%, though percentage agreement did not increase (0.189; *p* < 0.001). PET discriminatory power was not affected by histological tumour subtype.

**Conclusions:**

The results of our study indicate that PET might be a useful test for examination of the mediastinum in lung cancer patients. Its high NPV suggests that the absence of mediastinal uptake could be used to proceed to surgical treatment without the need for further tests or examinations. Nevertheless, studies directly aimed to answer this specific question are needed.

## 1. Introduction

Lung cancer is a major health problem in the 21st century, ranking as the second most common cancer in men after prostate cancer and the fourth most common cancer in women after breast, ovarian, and colon cancer. Moreover, it is the leading cause of cancer-related mortality in the Spanish population [1].

Lung cancer staging is essential information with a view to the treatment and prognosis of the disease [2, 3]. The disease tends to give rise to local metastasis of intrathoracic lymph nodes, both hilar and mediastinal, rendering it necessary to have methods that are capable of arriving at an accurate diagnosis for correctly planning treatments and thus modify the final prognosis [4]. Such methods are, in great measure, going to depend on the extent of the disease. Currently, the most widely used tool for mediastinal staging is the most recent version of the tumour, node, and metastasis (TNM) staging system, i.e., the 8th edition of the TNM published in 2016 by the International Association for the Study of Lung Cancer (IASLC) [2]. As lymph node mapping can vary with local societies' practice guidelines, there can be differences in staging. In 2009, the IASLC published its thoracic lymph node map, which is the one currently accepted for lung cancer staging [5].

At present, computed axial tomography (CT) and positron emission tomography (PET) are the key imaging tests for diagnosis of the extent of lung cancer. The utility of other mediastinal staging techniques, such as magnetic resonance [6], are being assessed, though as of now the leading clinical guidelines do not recommend their standardized use [7].

CT represents an advance in the study of the disease and has become the most widely used noninvasive test for evaluation of the mediastinum [8]. A proper anatomical examination makes it possible to ascertain the localization, number, and size of lymph node stations, whether they exhibit signs of invasion, and their relationship with adjoining structures according to the IASLC lymph node map [4]. The most commonly used criterion for classifying a lymph node as pathological is a short axis diameter ≥1 cm [8–10]. A systematic review found that this cutoff point ensures a sensitivity (S) and specificity (SP) of 57% and 82%, respectively; and its positive predictive value (PPV) and negative predictive value (NPV) are 62% and 87%, respectively, in examinations which indicate a prevalence of mediastinal metastasis of over 30% [11]. Even so, this test is not error free (false positives (FPs) and negatives (FNs)) and in view of such limitations should therefore not be considered definitive [12].

PET has amounted to an advance in mediastinal staging. Having less anatomical power than CT, it is a test that focuses on tumour metabolism and is thus able to ascertain whether there is mediastinal involvement or distant metastasis. The most used radiopharmaceutical is fluorine-18 2-fluoro-2-deoxy-D-glucose (18-FDG), which accumulates in greater quantities in cells with a more active metabolism, such as tumour cells [13]. PET results are interpreted using a quantitative system that calculates the maximum standardized uptake value (SUV_max_). While there are still no standardized values, it is nevertheless generally accepted that an SUV_max_ of over 2.5 is pathological [14, 15], though a lower SUV_max_ (FN) would not rule this out (bronchioloalveolar carcinoma, necrosis, etc.) [13]. For prevalence of metastatic mediastinal disease of 28%, PET sensitivity, specificity, and positive and negative predictive values surpass those of CT (80%, 88%, 75%, and 91%, respectively) [8]. On the other hand, the main limitation of PET lies in the possibility of false positives, the main causes of which are considered to be inflammatory alterations, infectious processes, anthracosis, and granulomatosis [16].

The year 2001 saw the commercial launch of imaging systems that combine the two tests (PET and CT) and have since come to replace the original PET systems [17]. Although a 2003 paper [18] suggested that the fusion of CT and PET images was superior to those produced by the two tests individually, no further analyses have been conducted in this respect and meta-analysis data would not seem to corroborate this finding.

Accordingly, the designated aim of this study was to ascertain the usefulness of PET in the examination of hilar and mediastinal lymphadenopathies in patients with suspicion of lung cancer, calculate its S, SP, PPVs, and NPVs, establish the best SUV_max_ cutoff point, compare the test's usefulness to that of CT, and determine whether histological tumour type influences these results.

## 2. Materials and Methods

### 2.1. Study Design

We conducted a retrospective study on a group of patients who were referred to a monographic lung cancer unit at the Santiago de Compostela Clinical University Teaching Hospital over a period of three years and diagnosed with resectable non-small-cell lung cancer (NSCLC) without evidence of distant metastasis. This health facility is a tertiary hospital equipped with the full range of radiological tests and pneumological techniques used in mediastinal staging. All patients underwent a CT (*General Electric Multislice LightSpeed scanner*, 5 mm collimation) and a PET scan (*General Electric Medical Systems Advance NXi* type tomography) for radiological characterization of adenopathies and finally a histological examination (biopsy or cytology) by surgically invasive (mediastinoscopy) or nonsurgically invasive mediastinal procedures (endobronchial ultrasound-guided needle aspiration/EBUS and echoendoscopy/EUS), in order to arrive at a definitive diagnosis. In view of the fact that each patient might present with various lymph node stations that were pathological in appearance, these were dealt with separately for analysis purposes, with lymph nodes—rather than patients—being used as the study unit. Lymph node stations that had not been examined by both imaging tests (PET and CT) or had not undergone histological examination were excluded from the analysis.

### 2.2. Study Variables

We studied the patients' demographic characteristics (age, sex, and histology of each lymph node station). With regard to the imaging tests analyzed, lymph node size was ascertained on the basis of CT and EBUS and SUV_max_ on the basis of PET. The “*N*” component was established according to the lymph node characteristics for each of the tests performed, using the TNM 8th edition and the IASLC lymph node map. The cutoff points used to classify adenopathies as pathological were short axis diameter ≥1 cm on CT and/or SUV_max_ > 2.5 on PET. For the different S, SP, PPV, NPV, and diagnostic accuracy values, the histological result was used as the gold standard. Analyses were performed by subgroup, taking into account the final histological diagnosis of each lymph node station.

### 2.3. Statistical Analysis

All the variables were recorded in a database and analyzed by means of the SPSS v22 computer software programme. A descriptive analysis was performed, with categorical variables expressed as absolute and relative frequencies and continuous variables expressed as means, medians, and ranges. S, SP, PPVs, and NPVs were calculated with the usual formulas. The degree of agreement between tests was evaluated by the weighted kappa analysis. Results were deemed significant at *p* < 0.05. To obtain the best SUV_max_ cutoff point, we calculated the area below the curve. All calculations were performed using the SPSS v22 programme.

## 3. Results

A total of 128 patients were analyzed: this broke down as 106 men (82.8%) and 22 women (17.2%), median age 67 years, and 54 (42.2%) of whom were active smokers. All patients underwent mediastinal staging by means of imaging tests (CT and PET), plus a histological examination. In all, 203 lymph node stations were completely studied: of this total, the most studied station was the right paratracheal lymph node (station 4R) (63; 31%), followed by the subcarinal lymph node (station 7) (62; 30.5%). The stations studied had a mean size, as measured by CT, of 14.5±8 mm, with the mean size as measured directly by EBUS being larger (15.5 ± 7.9 mm). The mean SUV_max_ was 6.4 ± 6.3. Taking into account the parameters established for classifying lymph nodes as pathological by CT and PET, 90 (44.3%) and 153 (75.4%) lymph nodes were considered pathologically suspect by CT and PET, respectively. After histological analysis, 58 (28.6%) lymph node stations exhibited metastasis of NSCLC, with the most frequent types being adenocarcinoma (35; 17.2%) followed by epidermoid carcinoma (23; 11.3%). A total of 48 lymph nodes (82.8%) were diagnosed by EBUS, 5 (8.6%) by mediastinoscopy, and 5 (8.6%) by surgery. In the remaining stations studied (145; 71.4%), there was no evidence of metastasis of NSCLC in the surgical piece. The main characteristics of the sample are summarized in Table 1.

The results obtained by routine mediastinal staging techniques (CT and PET) were compared with biopsy results, in order to ascertain whether these techniques had diagnosed each of the lymph node stations as benign or malignant, and thus be able to determine the usefulness of PET in mediastinal lymph node staging of NSCLC. Analysis of all the patients as a whole showed that CT and PET proved to be highly sensitive but not overly specific when compared with histology. Their sensitivity was 0.89 and 0.95, respectively, and their specificity was 0.40 and 0.37, respectively. The PPVs were low (0.49 in both cases), and the NPVs were considerably high (0.85 and 0.92 respectively). Percentage agreement between each scan and biopsy was low for diagnosis of each lymph node station (0.207 and 0.241, respectively; both *p* < 0.001) (Table 2).

**Figure 1:**
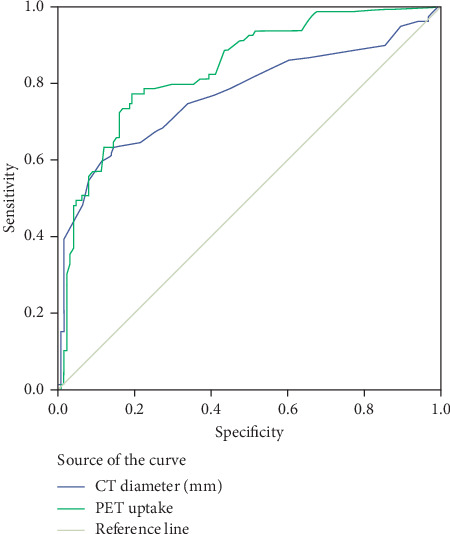
ROC curves. Diagonal segments are produced by ties.

On specifying which PET SUV_max_ afforded the greatest diagnostic accuracy from a statistical point of view, the area under the ROC curve was calculated as being 0.84 (Figure 1). Bearing in mind the fact that the value ensuring highest sensitivity and specificity for PET was an SUV_max_ of 0.5, the sample was analyzed again using this value as the cutoff point for classifying lymph nodes as pathological. In this case, PET sensitivity and NPV were 1 in both cases, and specificity and PPV were 0.24 and 0.46, respectively. Percentage agreement between PET (using an SUV_max_ cutoff point of 0.5) and histology was low for diagnosis of each lymph node station (0.189 (*p* < 0.001)) (Table 2).

With the aim of ascertaining whether histology of the primary lesion (adenocarcinoma versus some other histology) influenced the PET result in the examination of the mediastinum, an ROC curve analysis was performed. The values obtained were 0.46 and 0.40 for CT and PET, respectively, with no statistically significant differences in evidence, regardless of histological primary lesion type (*p*=0.382 and *p*=0.20, respectively).

## 4. Discussion

This study found PET to be a very sensitive test and with a high NPV in the examination of hilar and mediastinal lymphadenopathies in patients with suspicion of lung cancer, outperforming CT in terms of results. These values increase when a lower SUV_max_ is used (SUV_max_ 0.5), thereby reducing the number of FNs. No differences were found in uptake results by reference to the histology of the primary lesion.

The main clinical guidelines recommend the performance of a PET or PET/CT scan in patients diagnosed with NSCLC, save where the presence of metastasis has been detected on diagnosis [19]. The goal of a PET scan is to be able to rule out the presence of metastatic disease not evident in previous examinations and ensure the correct staging of locoregional lymph nodes, so as to plan the most appropriate treatment for each case in line with the results. This study confirms this usefulness but indicates that there might be a benefit according to the uptake used.

PET provides information on areas with greatest metabolic activity. In most cases, this activity is due to tumoral or metastatic disease, to infectious or inflammatory activity, in all cases producing greater cellular glucose uptake [16, 20]. While there is no universally accepted criterion for establishing lymph node positivity by PET or PET/CT, the most widely used criterion is qualitative or visual, whereby lymph node uptake is compared to uptake regarded as basal in the mediastinum [21]. Insofar as semiquantitative criteria are concerned, the most widely accepted value as cutoff point is SUV_max_ > 2.5, thanks to its high specificity and NPV [14].

Our study obtained an S of 95% and an SP of 37% for PET in lymph node staging of NSCLC. Comparison of both these values to the data yielded by a recent meta-analysis [14, 15, 22] shows that, whereas SP is lower (79.4%–90%), S is substantially higher (67%–81.3%). This finding may come as a surprise, bearing in mind that it pertains to a tuberculosis-endemic region [23], where PET sensitivity for detection of lymph node metastasis is reportedly lower [24].

The PPV was low (49%), due to the high proportion of false positives. Aside from tumours, smoking-related inflammation, infections, anthracosis, and necrotizing granulomatosis, among other causes, lead to increased uptakes in the PET scan, though they do not usually result in such a high SUV_max_ value [16]. It is for this reason that the different guidelines recommend histological confirmation of lymph nodes with positive uptake on PET, with minimally invasive techniques being of choice [25]. Furthermore, the NPV is high (92%) when an SUV_max_ cutoff point of 2.5 is considered and rises to 100% if this cutoff is 0.5. Faced with a negative PET result, one can proceed to surgery without the need for histological confirmation of the mediastinum, except in two cases described by the guidelines, i.e., central or large-sized tumours (T3) in which the risk of a hidden N2 is higher [25], something that is a relevant consideration.

The problem that arises in standard clinical practice is to define what level of metabolic activity is to be deemed negative or positive. Many studies have been conducted to date on this issue but with widely differing results. Apart from the factors associated with the PET model, namely FDG dosing and study design, there are others, such as (a) whether the analysis is performed on lymph nodes or patients, taking only the presence of mediastinal uptake into account, without differentiating between lymph node stations or individual lymph nodes; (b) the method used to ascertain the threshold of metabolic activity that is to be deemed positive. Some authors use a qualitative method with four levels of comparison in which the upper two levels are regarded as pathological [26], and others use the semiquantitative method. The most widely used SUV_max_ cutoff point is 2.5 [27], though others determine the SUV_max_ value using the ROC curve analysis for the best S and SP relationship, generally obtaining values higher than 2.5 [28, 29]; (c) whether or not to include hilar lymph nodes. Since their presence has prognostic connotations but does not modify the therapeutic attitude, some authors solely study the mediastinal lymph nodes; and (d) the pulmonary adenocarcinoma rate. Some subtypes, such as lepidic adenocarcinoma, on experiencing slow growth and lower metabolic activity, can give rise to FNs on PET [26].

Our study, which analyzed both hilar and mediastinal stations individually, observed that the best relationship between PET sensitivity and specificity (100% and 24% respectively) was obtained using an SUV_max_ value of 0.5. The PPV was 46% and the NPV was 100%, with a percentage agreement with histology of 0.189 (*p* < 0.001). The number of FPs (94/203; 46.3%) was mainly due to the presence of lymph node anthracosis, nonspecific inflammations, and non-necrotizing granulomatous inflammation. As matter of relevance, the NPV should be increased in order to minimize false negatives to the maximum, at the cost of having a higher number of false positives in this instance. This makes it possible to reduce underdiagnosis of metastatic nodules in lymph node staging and ensure that patients become candidates for the most appropriate treatment in each case.

On using the usual SUV_max_ cutoff point for examination of lymph node stations, S and NPV were reduced at the expense of an increase in FNs (from 0 to 4). PET sensitivity and specificity values were 95% and 37%, with PPVs and NPVs of 49% and 92%, respectively. Percentage agreement with histology in this case was 0.241 (*p* < 0.001).

Chest CT was compared with PET, employing the most widely used cutoff point in the literature to discriminate between normal lymph nodes and lymph nodes with suspicion of metastatic infiltration (short axis diameter ≥1 cm). CT displayed a lower S, NPV, and percentage agreement with histology than did PET.

Lastly, we evaluated the influence of primary tumour histology. In our series, the most frequent tumour was adenocarcinoma (30% of cases). A meta-analysis of 45 PET-CT studies [15] showed that the difference between histological subtypes could influence the results of mediastinal staging (the heterogeneity of PET being directly proportional to the number of adenocarcinomas). In our study, however, no significant differences were found between patients with adenocarcinoma versus the remaining histologies (*p*=0.20).

Our study's main limitations are described below. Firstly, we excluded patients with metastatic disease and thus not requiring mediastinal staging. This implies an underestimate of PET sensitivity and diagnostic accuracy in the detection of lymph node metastases, thus barring these data from being extrapolated to a general group of patients with lung cancer. Secondly, another perceived limitation was the delay between the tests performed on patients (CT, PET, and histological analysis). In some cases, the period that elapsed between the start and end of the examination exceeded one month and might have entailed changes in the tumour. Thirdly, the study's retrospective nature may entail risk of errors or biases in the data obtained; and lastly, influence was not assessed by histological type or by separate analysis of mediastinal or hilar lymph nodes. Among the study's advantages is the fact that all the lymph nodes analyzed featured a PET, CT, and biopsy, a factor that offsets its modest sample size.

In conclusion, this study indicates that PET is a test of great utility for examination of hilar and mediastinal adenopathies in patients with lung cancer. Its high NPV suggests that the absence of mediastinal uptake would make it possible to proceed to surgical treatment without the need for more studies, though there might be exceptions to such a claim.

## Figures and Tables

**Table 1:** Description of the sample: distribution by age, sex, smoking habit, mean nodule size, and characteristics of punctured lymph node stations.

	Age	Nodule size (mm)		SUV PET	Sex	Lymph node stations						Smoking habit	
		CT	EBUS			Punctured		Diagnosis		Method					
M	66	14.5	15.5	7	106 MEN (52.5%)	2R	2 (1%)	EC	23 (11.3%)	EBUS	48 (82.8%)	AS	54 (42.2%)
ME	67	11	14	4.7	22 WM (10.8%)	4R	63 (31%)	AC	35 (17.2%)	MT	5 (8.6%)	ES	43 (33.6%)
SD	10.2	8.1	7.8	11.1		4L	34 (16.8%)	MC	18 (8.9%)	S	5 (8.6%)	NS	17 (13.3%)
						7	62 (30.5%)	LPH	1 (0.5%)			ND	14 (10.9%)
						10R	8 (3.9%)	SC	7 (3.4%)				
						10L	7 (3.4%)	TB	8 (3.9%)				
						11R	16 (7.9%)	NLN	111 (54.7%)				
						11L	11 (5.4%)						

AC, adenocarcinoma; C, surgery; EC, epidermoid carcinoma; MC, microcytic carcinoma; SD, standard deviation; EBUS, endobronchial ultrasound-guided needle aspiration; ES: ex-smoker; AS, active smoker; NLN, normal lymph node; LPH, lymphoma; M, mean; ME, median; MT, mediastinoscopy; WM, women; ND, no data; NS, never smoker; PET, positron emission tomography; SC, sarcoidosis or sarcomatoid reaction; TB, tuberculosis; CT, computed tomography.

**Table 2:** CT and PET validity parameters versus histology (gold standard), using lymph nodes as the study unit.

A	Biopsy +	Biopsy −	Total

CT +	70	74	144
CT −	9	50	59
Total	79	124	203
PET +	76	78	154
PET −	4	45	49
Total	80	123	203

B	Biopsy +	Biopsy −	Total
PET +	79	94	173
PET −	0	30	30
Total	79	124	203

C	CT	PET A	PET B
S	0.89	0.95	1
SP	0.40	0.37	0.24
PPV	0.49	0.49	0.46
NPV	0.85	0.92	1
Percentage agreement and *p*	0.207 (*p* < 0.001)	0.241 (*p* < 0.001)	0.189 (*p* < 0.001)

(A) Preestablished cutoff points: positive CT if short axis ≥10 mm and positive PET if SUV >2.5. (B) ROC curve cutoff points: positive CT if short axis ≥2.5 mm and positive PET if SUV >0.5. (C) Sensitivity, specificity, predictive values, and percentage agreement for each PET cutoff point.

## Data Availability

The data used to support the findings of this study are available from the corresponding author upon request.
